# Vitamin D as a candidate host susceptibility factor in sugar-driven oral dysbiosis

**DOI:** 10.3389/froh.2026.1850800

**Published:** 2026-08-11

**Authors:** Moldir Turaliyeva, Nuraly S. Akimbekov, Ilya Digel, Adil Y. Yerezhepov, Mohammed S. Razzaque

**Affiliations:** 1 Department of Biotechnology, M. Auezov South Kazakhstan University, Shymkent, Kazakhstan; 2 Department of Biotechnology, Al-Farabi Kazakh National University, Almaty, Kazakhstan; 3 Ecology Research Institute, Khoja Akhmet Yassawi International Kazakh-Turkish University, Turkistan, Kazakhstan; 4 Institute for Bioengineering, Aachen University of Applied Sciences, Jülich, Germany; 5 Department of Medical Education, School of Medicine, University of Texas Rio Grande Valley (UTRGV), Edinburg, TX, United States; 6 Faculty of Medicine, Universiti Kuala Lumpur Royal College of Medicine Perak (UniKL RCMP), Ipoh, Perak, Malaysia

**Keywords:** dental caries, ecological plaque hypothesis, host susceptibility, oral dysbiosis, oral microbiome, periodontal inflammation, plaque biofilm, public health policy

## Abstract

**Introduction:**

Sugar-sweetened beverages (SSBs) represent a modifiable dietary factor that promotes oral dysbiosis by supplying fermentable sugars and generating low-pH conditions that favor acidogenic and acid-tolerant biofilms. Vitamin D plays a biologically significant role in oral host defense through regulation of the epithelial barrier, induction of antimicrobial peptides, immune modulation, inflammatory control, and mineral metabolism. However, the extent to which vitamin D modifies the SSB -oral microbiome -disease pathway in humans remains unclear.

**Methods:**

This narrative-conceptual review employed structured evidence mapping. Relevant literature was identified through PubMed/MEDLINE, Scopus, and Web of Science Core Collection, with additional sources obtained via citation and hand searching. Evidence was categorized into predefined domains: SSB/free-sugar exposure and oral outcomes; sugar exposure and oral microbiome characteristics; vitamin D status and oral health outcomes; vitamin D-related host-defense mechanisms; vitamin D and human oral microbiome evidence; and proposed SSB × vitamin D interaction pathways. A PRISMA-ScR-style flow diagram documented the process of evidence identification and selection, resulting in the inclusion of 90 sources. Evidence interpretation followed a tiered approach.

**Results:**

The most robust evidence indicates an association between frequent SSB/free-sugar exposure and caries-related outcomes. Human microbiome studies suggest that high sugar intake may be linked to dysbiotic shifts, although results differ depending on sampling site, study design, and analytical methods. Evidence connecting vitamin D to caries and periodontal outcomes is suggestive but remains heterogeneous. Direct human evidence relating 25(OH)D status to oral microbiome trajectories or SSB × vitamin D interaction models is limited. Mechanistic studies support biological plausibility but do not establish causality in humans.

**Discussion:**

Vitamin D should be considered a candidate susceptibility factor and a potential proxy marker for broader behavioral, socioeconomic, metabolic, and inflammatory risk environments. Future longitudinal studies are needed to jointly assess SSB exposure, standardized 25(OH)D levels, plaque-site microbiome trajectories, confounders, and incident oral outcomes. Current practice recommendations continue to emphasize reducing SSB consumption, encouraging non-sugary alternatives, promoting fluoride use, maintaining oral hygiene, and supporting preventive dental care.

## 1 Introduction

Oral diseases are among the most common non-communicable conditions worldwide, with caries and periodontal disease driving major disability and health-system costs (1–3). Although preventable, they persist along strong social gradients shaped by inequities and commercial forces that influence exposure to sugar-rich products (4–6). Understanding how diet drives disease, and why some individuals remain resilient under similar exposures, is essential for advancing precision prevention and public health policy. Caries and many inflammatory oral diseases involve biofilm-mediated, ecology-driven processes (7, 8). The oral microbiome typically exhibits dynamic stability supported by saliva (flow and buffering), epithelial barriers, and immune surveillance (9–12). Disease develops when recurrent environmental pressures disrupt equilibrium, selecting microbial communities with host-damaging metabolism and proinflammatory signaling, resulting in oral dysbiosis. Disease emerges when recurrent environmental pressures repeatedly disrupt this, favoring communities with host-damaging metabolic outputs (e.g., sustained acidification) and proinflammatory signaling, e.g., oral dysbiosis (7–9). The ecological plaque hypothesis formalizes this view by emphasizing that sustained habitat shifts, particularly repeated episodes of low pH, remodel plaque composition and function, rather than a single new pathogen being sufficient (13).

Free sugars are among the most consistent ecological drivers of oral dysbiosis (14, 15)**.** Fermentable carbohydrates fuel acidogenic metabolism within dental biofilms, and repeated acidification suppresses acid-sensitive organisms while aciduric taxa are selected. This shift reduces community diversity and increases the risk of enamel demineralization (16–19). Contemporary sequencing studies reinforce classic caries ecology by showing that caries-associated states arise from consortia of organisms and metabolic pathways that promote acid production, acid tolerance, and extracellular-polysaccharide-driven biofilm stability rather than from any single universally required cariogenic species (16).

Sugar-sweetened beverages (SSBs) are a particularly important form of dietary exposure because they are often consumed frequently throughout the day, especially between meals, and high concentrations of rapidly bioavailable free sugars are delivered (15, 20, 21). Systematic reviews and meta-analyses consistently associate higher SSB intake with greater caries risk and a greater likelihood of erosive tooth wear; dose–response patterns have been reported for caries (20, 22–24). Prospective studies in adults similarly indicate that a higher frequency of daily SSB consumption is associated with a greater increase in caries, even among individuals using fluoride toothpaste, suggesting that fluoride does not fully offset repeated sugar challenges (21). Additionally, because many beverages are acidic and/or chelating, the SSB matrix can intensify ecological stress through both substrate provision and direct pH disturbance (25, 26). Notably, identical sugar exposures do not yield uniform microbial or clinical outcomes (27, 28). Comparative studies of diet and microbial profiles frequently reveal substantial interindividual heterogeneity in plaque community responses, likely reflecting differences in baseline microbiome states, salivary buffering, enamel susceptibility, fluoride exposure, and immune tone (10).

Vitamin D is a biologically plausible host susceptibility factor in individuals exposed to sugar-driven oral dysbiosis (29). In addition to its classical endocrine functions in calcium and phosphate homeostasis, vitamin D exerts broad immunomodulatory actions relevant to epithelial barriers, antimicrobial defense, and inflammatory regulation at mucosal surfaces (30–36). Because direct human evidence linking vitamin D status to oral microbiome composition and longitudinal change remains limited, the proposed moderation pathway should be interpreted as hypothesis-generating and should not be taken as evidence that vitamin D supplementation alters the oral microbiome or offsets high SSB exposure.

Recent evidence on the subgingival microbiome and metabolic dysfunction extends this host susceptibility framework beyond vitamin D. Nibali et al. reported that subgingival microbial profiles are associated with metabolic dysfunction and metabolic-associated host genetic variants, suggesting that oral microbial ecology reflects systemic metabolic and genetic factors in addition to local dietary exposures (37, 38). Although these studies focus on subgingival periodontal niches rather than SSB-driven supragingival caries ecology, they highlight the importance of interpreting the SSB–microbiome–disease axis within a broader host metabolic and genetic context.

This review integrates diet–microbiome ecology with host biology to examine how frequent sugar exposure, particularly from SSBs, drives oral dysbiosis and how vitamin D status may influence the transition from reversible ecological shifts to persistent disease. Vitamin D is presented as a candidate susceptibility modifier to guide future research and prioritize key measurements, not as evidence that vitamin D–targeted interventions currently improve SSB-related oral microbiome or disease outcomes. Importantly, vitamin D status may also reflect broader risk environments, as low 25(OH)D commonly co-occurs with poorer diet quality, limited sun exposure, increased adiposity, socioeconomic disadvantage, heightened inflammatory burden, and reduced access to preventive care. Accordingly, this review treats biological susceptibility and proxy-marker explanations as competing, but not mutually exclusive, hypotheses requiring further study. The term “host susceptibility factor” is therefore used as a testable effect-modifier concept, rather than evidence that vitamin D has been shown to modify the SSB–microbiome–disease axis in humans.

## 2 Methods

### 2.1 Review design and reporting approach

#### 2.1.1 Primary review design

This manuscript is framed as a narrative-conceptual review supported by structured evidence mapping rather than a systematic or scoping review. The aim was to develop an integrative model linking SSB exposure, oral microbiome ecology, and vitamin D–related host susceptibility, not to estimate pooled effects. Search and selection procedures were documented transparently, but synthesis was organized conceptually and narratively because the evidence spans heterogeneous domains, including epidemiology, microbiome profiling, clinical interventions, and mechanistic *in vitro* and animal studies.

#### 2.1.2 Use of reporting frameworks

To avoid ambiguity, PRISMA-ScR was used only as a reporting aid for the literature-identification flow diagram, and JBI scoping review guidance informed broad evidence-mapping procedures (39–43). SANRA was used as a narrative-review quality checklist. These frameworks supported transparency but do not imply that the manuscript is a PRISMA 2020 systematic review with meta-analysis or a fully registered scoping review (44, 45). Accordingly, conclusions are presented as conceptual and hypothesis-generating where direct human evidence is limited.

### 2.2 Information sources

Searches were conducted across multiple scientific databases up to 26 May 2026 (final update) to capture both biomedical and interdisciplinary evidence, including newly published oral microbiome and vitamin D studies. The PubMed/MEDLINE, Scopus, and Web of Science Core Collection databases were searched. Additionally, reference lists of highly relevant reviews and key primary studies were screened (backward citation searching), and forward citation searching was used when feasible to identify newer studies that cited foundational work. For methodological transparency, the search record was used as an evidence-identification log rather than for statistical meta-analysis. The search results were exported to a reference manager, de-duplicated before screening, and supplemented by backward and forward citation searches of relevant reviews and primary studies. No publication-year restrictions were applied, allowing the inclusion of both seminal ecological and methodological studies and recent human microbiome and vitamin D research. During evidence synthesis, contemporary systematic reviews and human studies were prioritized when they addressed the same questions as older publications did.

### 2.3 Search strategy (concepts and key terms)

A structured, iterative strategy was built around three intersecting concepts:

1. Sugar exposure/SSBs: “sugar-sweetened beverage*,” “soft drink*,” “soda,” “carbonated beverage*,” “energy drink*,” “sweetened drink*,” “added sugar*,” “free sugar*,” and (when needed) broader diet terms such as “dietary sugars” combined with oral endpoints.
2. Oral microbiome and dysbiosis: “oral microbiome,” “salivary microbiome,” “dental plaque microbiome,” “supragingival plaque,” “subgingival,” “biofilm,” “dysbiosis,” “microbial ecology,” “cariogenic biofilm,” plus sequencing terms (“16S rRNA,” “metagenomic*,” “amplicon sequencing”).
3. Vitamin D and host susceptibility: “vitamin D,” “25-hydroxyvitamin D,” “25(OH)D,” “cholecalciferol,” “calcitriol,” “vitamin D receptor,” “VDR,” “supplementation,” and immune-related terms relevant to host–microbe interactions (e.g., “innate immunity,” “antimicrobial peptide*,” “inflammation”).

The search strings incorporated Boolean logic (AND/OR), database-controlled vocabulary where available (such as MeSH terms in PubMed), and truncation (for example, microbiom*) to capture variant spellings. Owing to differences in terminology across fields such as nutrition, dentistry, and microbiome science, the strategy was refined following an initial screening of sentinel papers to ensure sensitivity.

The reproducible master logic used across databases was as follows: (SSB/free-sugar terms) AND (oral microbiome/biofilm/oral-disease terms) AND [vitamin D/25(OH)D/VDR terms]. Because direct three-way human evidence is expected to be sparse, additional pairwise searches were also conducted for (1) SSB/free sugar plus oral microbiome or oral disease; (2) vitamin D/25(OH)D plus oral microbiome or oral disease; and (3) vitamin D/VDR plus gingival immunity, antimicrobial peptides, epithelial barrier function, or inflammation. Database-specific versions used controlled vocabulary where available (e.g., MeSH in PubMed/MEDLINE) and title/abstract keywords in Scopus and the Web of Science Core Collection. This broader strategy was used to identify the distinct evidence streams required for conceptual synthesis, while the eligibility criteria below determined which records were retained.

Representative search syntax included combinations of the following strings: “sugar-sweetened beverage*” OR “soft drink*” OR soda OR “carbonated beverage*” OR “energy drink*” OR “free sugar*”; “oral microbiome” OR “dental plaque microbiome” OR “salivary microbiome” OR biofilm* OR dysbiosis OR caries OR periodontal; and “vitamin D” OR “25-hydroxyvitamin D” OR 25(OH)D OR calcitriol OR cholecalciferol OR VDR. Full-text papers were then categorized into evidence domains (SSB/oral outcomes, SSB/oral microbiome, vitamin D/oral outcomes, vitamin D/oral host-defense mechanisms, and contextual/policy evidence), which made explicit how evidence was identified across disciplines.

### 2.4 Eligibility criteria

Eligibility criteria were developed using the population-concept-context (PCC) logic recommended for scoping-style reviews.

- Population: Humans of any age (children, adolescents, adults). Mechanistic *in vitro* or animal studies were eligible when they directly informed the biological plausibility of vitamin D-immune pathways or sugar-driven biofilm ecology but were interpreted separately from human evidence. Non-human evidence was used to support biological plausibility and to frame hypotheses; conclusions about clinical applicability were anchored primarily in human studies.
- Concepts: Studies were eligible if they addressed at least one of the following:
  - SSB/free-sugar exposure in relation to oral microbiome/biofilm outcomes or oral disease outcomes;
  - Vitamin D status [typically serum 25(OH)D], intake, or supplementation in relation to oral health outcomes and/or oral microbiome features;
  - Host–microbe mechanisms relevant to vitamin D signaling, antimicrobial activity, mucosal/gingival immunity, or inflammation in the oral environment.
- Context: Community, clinical, and laboratory settings; any geographic region.

The outcomes of interest included (1) oral microbiome composition or function (alpha/beta diversity, taxa, functional pathways, acidogenic potential), (2) intermediate ecological measures (plaque pH dynamics when reported), and (3) clinical outcomes (caries indices such as dmft/DMFT, ICDAS where available, gingival indices, periodontal measures).

The exclusion criteria included non-oral microbiome studies (e.g., gut-only), papers lacking any measure of sugar/SSB exposure or vitamin D exposure/status when those were central to the review question, non-researched articles without analyzable data (unless used for background definitions), and studies where outcomes were not oral/microbial/ecological.

### 2.5 Study selection and data charting

**Figure 1:**
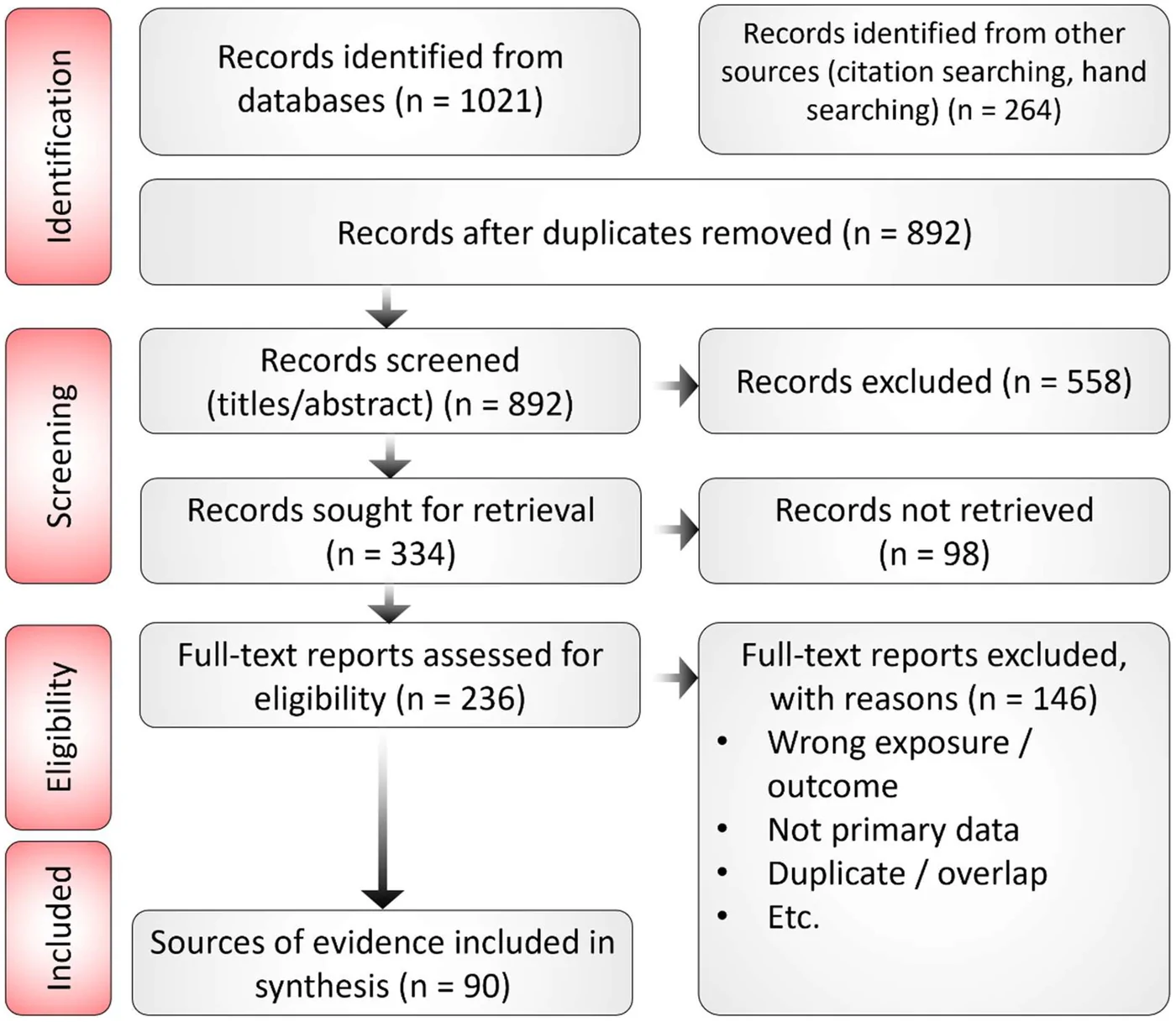
PRISMA-ScR-style flow diagram documenting the literature identification, screening, eligibility assessment, and inclusion process for the narrative-conceptual review.

Titles and abstracts were screened initially, followed by full-text assessment for potentially eligible papers. Reasons for exclusion at the full-text stage were recorded. The study selection process is summarized in a PRISMA-ScR-style flow diagram (Figure 1) to document the transparent identification, screening, eligibility assessment, and inclusion process; Figure 1 is used for reporting transparency rather than to claim a formal systematic review synthesis (40). In total, 1,285 records were identified, 892 records remained after duplicate removal, 236 full-text reports were assessed for eligibility, and 90 sources of evidence were included in the synthesis.

The identification and selection process was performed numerically as follows: 1,021 records were identified from the databases, and 264 records were identified from the citation/hand search; after duplicate removal, 892 records were subjected to title/abstract screening. At the title/abstract level, 558 records were excluded because they were clearly outside the review scope, leaving 334 reports to be retrieved. Ninety-eight reports were not retrieved, and 236 full-text reports were assessed for eligibility. A total of 146 full-text reports were excluded for incorrect exposure or outcome, the absence of primary data when primary evidence was needed, duplicate/overlapping datasets, or other scope-related reasons; 90 sources of evidence were retained for synthesis (Figure 1).

To clarify the selection logic, records were not required to address all three core elements (SSB exposure, vitamin D, and oral microbiome) simultaneously because such direct human studies are rare. Instead, records were included if they contributed directly to one of the predefined evidence domains needed to construct or test the conceptual model. Non-human or *in vitro* studies were retained only when they informed biological plausibility (for example, cariogenic biofilm ecology or vitamin D-mediated gingival/epithelial host defense) and were synthesized separately from human clinical or microbiome studies. The count of sources included in Figure 1 refers to evidence sources used in the synthesis; the bibliography may include a small number of additional reporting frameworks and methodological or contextual references that supported manuscript preparation but were not counted as primary sources of evidence.

A standardized data-charting form was used to extract the publication year, country, population characteristics, study design, exposure definitions (such as SSB frequency, added/free sugar metrics, beverage categories, and timing or sipping behavior if reported), vitamin D assessment method [serum 25(OH)D thresholds, intake instruments, supplementation dose and duration], oral sampling site (saliva, plaque, or subgingival), microbiome method (16S region, sequencing platform, bioinformatics pipeline), clinical outcomes, confounders controlled (including fluoride exposure, oral hygiene, socioeconomic factors, and total diet quality), and key findings. For each included record, the evidence domain, study design, population, exposure/outcome alignment, and reason for inclusion in the conceptual framework were charted so that the contribution of each source to the synthesis could be traced.

Vitamin D assessment was performed in detail, including the biomarker [total serum 25(OH)D], assay platform and calibration when reported (e.g., immunoassay vs. LC–MS/MS), units (nmol/L vs. ng/mL; 1 ng/mL = 2.496 nmol/L), sampling season/timing, and the study-specific thresholds used to define deficiency/insufficiency/sufficiency. Because intraassay variability and differences in guideline cutoffs can materially change the classification of “low” vitamin D status, the results were interpreted using within-study contrasts rather than assuming that absolute thresholds were comparable across studies (46–50).

### 2.6 Critical appraisal of the included studies

SANRA was used only as a reporting-quality check for the narrative review and was not treated as a risk-of-bias or certainty-of-evidence instrument. To improve interpretability across heterogeneous sources, we added a structured, domain-level evidence appraisal rather than applying a single study-level tool to all included designs. The appraisal was guided by the GRADE concepts and study-type appraisal principles used in the AMSTAR 2, ROBINS-I, and risk-of-bias frameworks (40, 43, 51–53). For each evidence stream, we considered study design, risk of bias, directness to the review question, consistency, temporality, confounding control, measurement validity [SSB exposure, oral sampling site, sequencing methods, 25(OH)D assay and cutoff], and applicability to the human oral microbiome or clinical outcomes. Certainty was categorized as higher, moderate, low, or very low/insufficient for the specific claim being made. This domain-level appraisal was used to calibrate the wording of conclusions and is summarized in a Table; it does not imply that the review generates pooled certainty estimates across all evidence domains.

### 2.7 Synthesis approach

The findings were synthesized narratively and conceptually rather than statistically. The organizing framework was specified *a priori*: (1) SSB exposure as an ecological pressure, (2) oral microbiome shifts and functional consequences, and (3) vitamin D as a plausible host susceptibility factor. Evidence was separated by study type (systematic review/meta-analysis, human observational study, intervention study, microbiome profiling study, and mechanistic *in vitro*/animal study) so that biological plausibility was not conflated with direct clinical evidence. Where direct human data were limited, statements were framed as hypotheses or future research priorities rather than practice-ready conclusions (39, 48, 50).

To avoid overextending broad conclusions across heterogeneous studies, synthesis statements were tiered by evidence type: findings from systematic reviews/meta-analyses and larger human observational studies were used for population-level associations; smaller human microbiome studies were treated as associative and exploratory; and animal/*in vitro* studies were used only to support mechanistic plausibility. Consequently, claims about clinical practice were limited to better-supported SSB reduction evidence, whereas the vitamin D interaction model was presented as a research agenda requiring direct human testing. For this manuscript, a stricter evidence-tier “non-upgrading” rule was applied: mechanistic or experimental findings may strengthen plausibility, but they do not increase the proposed human oral microbiome effect unless supported by the human microbiome or clinical outcome data that measure the relevant pathway directly.

## 3 Conceptual framework

### 3.1 Ecological host susceptibility model

**Figure 2:**
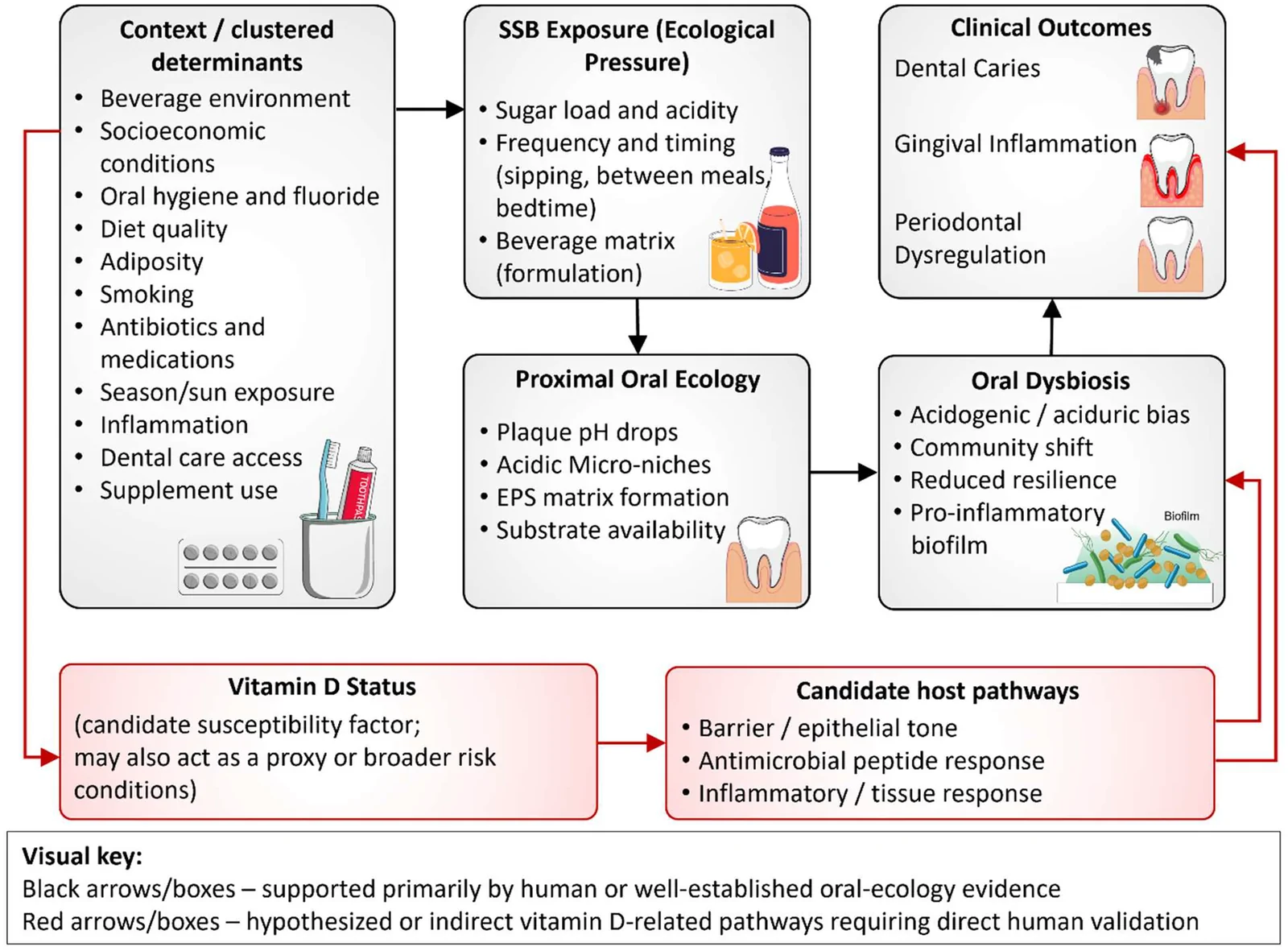
Ecological-host susceptibility model of sugar-driven oral dysbiosis. The solid black arrows/boxes denote pathways supported primarily by human or well-established oral-ecology evidence, whereas the dashed gray arrows/boxes denote hypothesized vitamin D-related pathways or indirect inferences that require direct human validation. The figure should be interpreted as a conceptual, hypothesis-generating framework rather than a validated causal model.

This review adopts an ecological, host-microbe framework in which SSBs act as a recurring environmental perturbation to oral biofilms, while vitamin D status affects host susceptibility and resilience. Contemporary models of oral disease emphasize that caries and other biofilm-mediated conditions rarely reflect a single pathogen; instead, they arise when local habitat conditions repeatedly shift microbial community structure and metabolic output toward acidogenic/aciduric and inflammatory phenotypes (ecological plaque hypothesis) (8, 13) (Figure 2).

The oral microbiome is highly biogeographic, with niche-specialized communities that are structured by saliva flow, shear forces, oxygen tension, pH, and the availability of host- and diet-derived substrates (10–12). SSB exposure alters this habitat through rapid, repeated decreases in plaque pH (the “Stephan curve” concept), increased availability of fermentable carbohydrates, and, depending on beverage formulation, additional erosive or chelating effects from acids (16, 23, 54). Accordingly, the framework incorporates a broader metabolic–genetic host layer. Vitamin D is treated as the focal immunonutrition factor in this review, but it is not the only host correlate of oral microbial ecology. Evidence linking subgingival microbial composition with metabolic dysfunction and metabolic-associated genetic variation indicates that baseline host metabolic and genetic background may shape the periodontal microbiota before, or alongside, beverage-related ecological pressures (37, 55). This helps position the present review within a wider oral-systemic framework while keeping the vitamin D interaction hypothesis appropriately focused and testable.

To avoid privileging a biological mechanism over equally plausible social and behavioral explanations, the framework also treats 25(OH)D as a potential proxy variable. Under this interpretation, observed vitamin D-oral health associations may reflect clustered exposures (e.g., diet, sugar consumption, hygiene, fluoride access, sun exposure, adiposity, and inflammation) that jointly shape oral microbial ecology and disease risk. To reduce the risk of overinterpretation, Figure 2 now uses explicit visual conventions to distinguish evidence tiers: the SSB **→** ecological pressure **→** dysbiosis **→** oral outcome pathway is shown with solid black arrows because it is grounded in human and ecological evidence, whereas vitamin D-related links are shown with dashed gray arrows and shaded boxes because they remain candidate pathways supported mainly by mechanistic or indirect evidence. This visual distinction is intended to reinforce that the model organizes current knowledge and uncertainties rather than depicting a validated human mechanism.

This conceptual model positions SSB exposure as repeated ecological pressure that perturbs the oral biofilm environment and promotes oral dysbiosis. Vitamin D status is framed as a host susceptibility factor that may modify (1) the likelihood that dysbiosis consolidates under sugar challenge and (2) the tissue/inflammatory response to dysbiotic biofilms. Because direct human evidence linking vitamin D status to oral microbiome composition remains limited, the vitamin D moderation components are presented as testable hypotheses supported by mechanistic plausibility rather than established clinical effects. The model begins with upstream determinants (e.g., socioeconomic context, beverage availability/marketing, dietary patterning, oral hygiene resources, fluoride exposure, dental care access, season/outdoor time, supplement use, antibiotics) that shape both SSB intake and vitamin D status and may confound the observed associations.

To make the level of inference explicit, the better-supported diet–biofilm–clinical outcome pathway is distinguished from the proposed vitamin D moderation pathway. The latter is represented as a dashed, hypothesis-generating link that requires direct testing with human 25(OH)D, site-specific oral microbiome, and longitudinal outcome data. SSB exposure is defined by both dose and consumption pattern, including sugar concentration and volume, frequency of intake, between-meal sipping, and bedtime consumption, as well as beverage matrix characteristics such as acidity and other formulation features. These exposures induce proximal oral ecological changes, such as repeated decreases in plaque pH, increased demands on salivary buffering, and increased selection for acidogenic and aciduric microbial functions. Biofilm-level processes, including extracellular polysaccharide matrix development and the formation of diffusion-limited microniches, further amplify localized low-pH conditions at the tooth surface, thereby reinforcing dysbiotic stability.

The microbial ecological response is characterized by alterations in community structure and function, including reduced resilience, shifts in taxa networks, increased carbohydrate fermentation capacity, and enhanced acid stress responses. Downstream, dysbiosis contributes to clinical outcomes, primarily dental caries, through sustained demineralizing conditions and gingival inflammation or early periodontal dysregulation through biofilm–host inflammatory interactions. These outcomes involve overlapping yet distinct biological pathways.

### 3.2 Vitamin D as a moderator of sugar-driven dysbiosis

Vitamin D is positioned in this framework as a host-factor that can modify (“moderate”) the biological consequences of similar SSB exposures. Mechanistically, vitamin D signaling via the VDR may support epithelial barrier integrity, tune immune responses, and promote antimicrobial peptide expression, thereby influencing inflammatory set points and the capacity to recover from perturbation (34, 35). However, most supporting evidence for these host defense pathways comes from mechanistic and non-human studies, and their net effects on human oral microbiome trajectories have not been established. Accordingly, the model is intended to support hypothesis generation and study design [e.g., testing SSB × 25(OH)D interactions] rather than justifying vitamin D supplementation as an oral microbiome intervention in current clinical practice.

### 3.3 Pathways to clinical outcomes and intervention leverage points

The conceptual model therefore links (1) SSB exposure features (frequency, timing, acidity, and co-ingredients), (2) ecological pressures within plaque/saliva (low pH, altered redox, and substrate pulses), (3) microbial functional shifts (greater acid production, extracellular matrix remodeling, and stress-response programs), (4) host responses (salivary buffering, enamel de/remineralization balance, and mucosal immunity), (5) outcomes, including caries lesion activity, erosive tooth wear, and periodontal inflammation (19, 56). At the population level, policies that reduce SSB affordability, availability, or marketing may be justified by existing evidence concerning the effects of sugar and public health, but this review does not claim that such policies operate through vitamin D-mediated microbiome modification unless that pathway is directly evaluated (57–60).

## 4 SSBs and sugar-driven oral dysbiosis

### 4.1 Defining “sugar-driven dysbiosis” in the oral ecosystem

Dental plaque is a complex, multispecies biofilm whose composition and activity are shaped by local habitat conditions and host factors such as saliva flow/buffering, mucosal barrier integrity, and immune monitoring (8, 10). Building on ecological plaque concepts, sugar-driven dysbiosis can be understood as a shift toward acidogenic and acid-tolerant functional profiles under repeated low-pH episodes rather than a single-organism model of disease (13).

Within this framework, “sugar-driven oral dysbiosis” describes a cycle in which fermentable sugars enter dental plaque, microbial metabolism generates organic acids, and plaque pH falls below the threshold that favors demineralization. Repeated episodes of acidification select for aciduric organisms and stress-response programs, reinforcing an environment that sustains low pH and accelerates enamel mineral loss (19). In parallel, the community can become enriched for extracellular polysaccharide (EPS) matrix production, enhancing biofilm retention and diffusion limitation, which further stabilizes acidic microenvironments (56).

### 4.2 Why SSBs are a distinctive ecological pressure

Systematic reviews have indicated that reducing free-sugar intake is associated with decreased caries occurrence and progression (15). SSBs are particularly significant because they are often consumed frequently between meals, deliver high sugar loads in a liquid form that rapidly permeates biofilms, and may co-deliver acids that lower the pH independently of fermentation (26). Evidence suggests that the frequent consumption of sugar-rich foods and beverages is linked to increased caries risk among children and adolescents, supporting upstream dietary policy approaches (24). Observational evidence linking SSB consumption to oral disease supports this ecological perspective. Meta-analyses and cohort studies generally report higher caries incidence or increases among individuals with greater SSB frequency, even in settings with fluoride toothpaste use (22). In parallel, acidic soft drinks and energy drinks are consistently implicated in erosive tooth wear, particularly when exposures are frequent or involve behaviors that prolong oral clearance (e.g., sipping over extended periods) (61). Mechanistically, sugar-driven dysbiosis involves not only increased acid production but also alterations in biofilm architecture and microenvironments. The EPS-rich matrix produced from sucrose can increase biofilm adhesion and create diffusion-limited niches where acids accumulate (56). Acid stress also activates adaptive bacterial responses (e.g., proton pumps, altered membrane composition, and DNA/protein repair systems) that increase survival at low pH, reinforcing aciduric community function (25). Table 1 summarizes key SSB exposure dimensions and the expected ecological pressures they impose on dental biofilms, providing a framework for interpreting microbiome findings across heterogeneous study designs.

**Table 1:** SSB exposure features and expected microbial ecological pressures.

SSB exposure feature	Typical operationalization (examples)	Expected ecological pressure in plaque/biofilm	Expected microbial ecological response (community/function)	Key references
High intake frequency	Servings/day; sugar exposures/day; “daily” vs. “<weekly”	Repeated plaque pH depressions; less time for pH recovery between challenges	Selection for aciduric + acidogenic traits; higher “acidogenic potential”; reduced resilience/homeostasis	(7, 13, 20, 21, 54)
Between-meal intake (snacking pattern)	Between-meal SSB episodes; “sipping” outside meals	Longer cumulative time under cariogenic conditions (more frequent pH drops, less buffering from meal-related saliva stimulation)	Greater persistence of low-pH niches; higher likelihood of dysbiosis consolidation	(13, 20, 21, 54)
Bedtime/low-salivary-flow intake	SSB at bedtime; intake during xerostomia/medication use	Slower clearance + buffering; prolonged low pH; prolonged sugar availability	Stronger selection for acid-tolerant communities; greater risk of sustained dysbiosis and demineralization pressure	(13, 62)
High sugar load per serving/per day	Sugar/serving; total g/day from beverages; “high-sugar” categories	More fermentable substrate → higher acid production; greater pH drop magnitude; more frequent acidification when combined with frequency	Functional shifts toward carbohydrate uptake/fermentation; potential enrichment of taxa common in caries-associated states (context-dependent)	(13, 15, 27, 54)
Sucrose-rich beverages (vs. non-sucrose sugars)	Sucrose as primary sweetener; exposure experiments	Sucrose supports EPS matrix production (biofilm architecture); diffusion limitation and acidic microniches	More stable cariogenic biofilms; stronger ecological “ratchet” toward dysbiosis; community imbalance	(56, 63)
Acidic beverage matrix (low pH/high titratable acidity)	Beverage pH; titratable acidity; cola/citrus-based drinks; energy drinks	Direct pH stress + repeated low-pH exposure (even apart from fermentation); may compound pH selection pressure	Selection for acid-tolerant organisms/functions; can reinforce low-pH ecology (and adds erosion risk)	(13, 20, 54)
Prolonged sipping duration	Time-to-finish; “grazing” over 15–60 min; multiple sips/hour	Extends time in an acidified microenvironment; repeated micropulses of substrate	Greater stability of low-pH niches; favors organisms with acid stress adaptation and persistent biofilm growth	(54)
Co-occurrence with overall high free-sugar diet	Dietary pattern scores; added/free sugar intake	Reinforces frequent substrate supply across the day; reduces ecological “recovery windows”	Broader, sustained dysbiosis risk; more consistent community-level functional shift	(15, 27, 28)
Observed microbiome signals in higher-sugar exposure studies	Diversity (*α*/*β*), taxa networks, functional pathways (16S/shotgun)	Detectable shifts in community structure linked to sugar exposure patterns	Often: lower richness/altered composition; caries-risk phenotypes may differ even at similar sugar intake (host/microbiome variability)	(28, 64)

### 4.3 Oral microbiome findings associated with high sugar or SSB exposure

Microbiome studies generally indicate that sugar-rich diets are associated with reduced oral microbial diversity and shifts in community composition, although reported taxa vary by study design and sampling site (16, 27). Across datasets, high sugar exposure is commonly associated with enrichment of acidogenic/aciduric organisms (including mutans streptococci and lactobacilli) and depletion of health-associated taxa that contribute to alkali generation or ecological buffering (18, 65). Interpretation of evidence linking SSBs to the oral microbiome depends on the site and method of microbiome measurement. Saliva sampling is convenient and may reflect a composite signal from multiple oral habitats, whereas supragingival plaque more directly captures the biofilm microenvironment where pH-driven selection occurs (10, 66). Harmonized sampling, metadata capture (e.g., time since last sugar exposure, oral hygiene behaviors), and analytic standards are therefore critical for causal inference and cross-study comparability (67, 68). Overall, the literature suggests that SSBs promote dysbiosis by repeatedly supplying fermentable sugars and, in many cases, adding an acid load. Outcomes vary with host susceptibility, including salivary buffering, fluoride exposure, enamel integrity, and immune factors such as vitamin D–VDR signaling (31).

## 5 Vitamin D biology is relevant to oral host defenses

Vitamin D is a pleiotropic immunonutrient; its status is assessed by 25(OH)D, which is converted to 1,25(OH)_2_D to regulate gene expression via VDR, influencing mineral metabolism, epithelial homeostasis, and immune function (30, 32, 34, 69).

### 5.1 Local activation and vitamin D signaling in oral tissues

A central question in oral host defense is whether oral tissues can generate active vitamin D locally, independent of systemic endocrine sources. Evidence supports local activation pathways in the gingiva, including the expression of vitamin D-metabolizing enzymes (e.g., 25-hydroxylase and 1*α*-hydroxylase/CYP27B1) in gingival epithelial cells and connective tissue cells. Exposure to 1,25(OH)_2_D can alter antibacterial and inflammatory responses in oral epithelial models, supporting an autocrine/paracrine model of vitamin D action at the mucosal interface (70–72).

### 5.2 Vitamin D and “host filtering”: antimicrobial peptides and innate defense

The regulation of antimicrobial peptides (AMPs) represents a key mechanistic link between vitamin D and mucosal defense (73–75). Foundational work has demonstrated that 1,25(OH)_2_D can directly induce antimicrobial peptide gene expression and that the human CAMP is a transcriptional target of VDR signaling (76, 77). Subsequent studies established a coherent axis linking innate immune activation to vitamin D pathway engagement: Toll-like receptor stimulation in human macrophages increases the expression of VDR and 1*α*-hydroxylase, resulting in cathelicidin induction and enhanced antimicrobial activity (78). In the oral cavity, the gingival epithelium functions as an active immune interface rather than a passive barrier. Studies have demonstrated that vitamin D induces innate immune defense mechanisms in gingival epithelial cells, including increased AMP activity and enhanced antibacterial defense *in vitro*. These findings support the hypothesis that sufficient vitamin D signaling may contribute to the “host filtering” of microbial communities by reinforcing epithelial antimicrobial capacity (79, 80).

### 5.3 Barrier integrity, epithelial homeostasis, and inflammatory Set points

The integrity of the oral epithelium is essential for host defense, as it influences microbial attachment, resistance to invasion, and immune–microbe interactions. Vitamin D/VDR signaling regulates epithelial proliferation and differentiation across various tissues. Specifically, in oral keratinocytes, vitamin D signaling affects proliferation and differentiation both *in vitro* and *in vivo*, suggesting a plausible mechanism through which vitamin D may support barrier maintenance and repair in the oral mucosa (81). Vitamin D is an immune modulator capable of influencing both innate and adaptive responses and of shaping the inflammatory tone, with effects that depend on the cell type and microenvironment (33). In oral-specific models, vitamin D signaling in gingival epithelial cells has been associated with reduced intracellular growth of periodontal pathogens (e.g., *P. gingivalis*) and with altered inflammatory outcomes in experimental systems (71, 82).

### 5.4 Mineral metabolism and tissue resilience under biofilm challenge

While dysbiosis represents an ecological imbalance, its progression to clinical disease is influenced by tissue resilience. The classical role of vitamin D in calcium–phosphate homeostasis provides a systemic foundation for the health of mineralized tissues and bone remodeling (83, 84). In periodontal models, dietary vitamin D restriction has been linked to increased gingival inflammation and alveolar bone loss in mice, indicating that adequate vitamin D levels may affect the response of periodontal tissue to microbial challenges as part of the broader host susceptibility landscape (71). In summary, vitamin D is biologically positioned to influence oral host defenses through several mechanisms: (1) local activation and VDR signaling in gingival tissues, (2) AMP-mediated antimicrobial filtering, (3) maintenance of epithelial barrier homeostasis and regulation of inflammation, and (4) support of mineral and bone-related tissue resilience. These pathways establish a mechanistic rationale for considering vitamin D as a modifier of sugar-driven dysbiosis rather than as an independent cause of oral disease.

## 6 Evidence synthesis

This section synthesizes evidence from four interconnected domains: (1) SSB exposure and its impact on oral ecological disruption and dysbiosis, (2) vitamin D status in relation to oral disease outcomes, (3) vitamin D effects on the oral microbiome and host-microbe mechanisms, and (4) the hypothesis that vitamin D affects susceptibility to sugar-driven dysbiosis and its sequelae. These collective findings align with an ecological framework in which SSBs exert repeated substrate and pH stresses on plaque biofilms, whereas vitamin D is thought to influence host filtering and inflammatory responses. The strength of evidence varies: it is robust for SSB-caries associations, moderate for SSB-erosion and SSB-microbiome links, suggestive but heterogeneous for vitamin D-caries and vitamin D-periodontal outcomes and limited for vitamin D-oral microbiome relationships. Accordingly, practice-relevant conclusions are the strongest for reducing SSB exposure, whereas vitamin D–microbiome links should be interpreted as provisional pending confirmation in well-designed human studies. For transparency, we label whether the findings are supported by meta-analyses/systematic reviews, primary observational studies, or mechanistic/experimental evidence in the subsections below. Evidence-boundary statement: No coherent body of longitudinal human studies currently demonstrates that vitamin D status affects the SSB microbiome–disease pathway. The framework therefore connects established evidence streams to formulate a testable interaction hypothesis rather than to claim an evidence-established mechanism. Evidence-tier hierarchy used in Sec. 6: To prevent different evidence streams from being read as equivalent, the synthesis below distinguishes five levels of inference:

*Tier 1*—established/direct human outcome evidence: systematic reviews/meta-analyses and prospective human studies linking SSB/free-sugar exposure to oral outcomes; this tier supports population-level prevention messages.
*Tier* 2—human vitamin D–oral outcome evidence: observational meta-analyses, clinical cohorts, and supplementation studies addressing caries or periodontal outcomes; this tier supports cautious association or adjunctive-treatment hypotheses but remains vulnerable to confounding, reverse causality, and assay/cutoff heterogeneity.
*Tier 3*—human oral microbiome evidence: human sequencing or metagenomic studies relating SSB/sugar exposure or vitamin D status to saliva or plaque communities; this tier is generally exploratory unless longitudinal, site specific, and outcome-linked.
*Tier 4—*mechanistic support: cell culture, epithelial, VDR/cathelicidin, *in vitro* biofilm, and animal evidence; this tier explains biological plausibility but is not treated as direct evidence that vitamin D modifies human oral microbial ecology.
*Tier 5*—speculative extension: the integrated SSB × vitamin D effect-modification model; this remains a falsifiable research hypothesis requiring direct human testing across exposure, 25(OH)D, oral microbiome, and clinical outcome measures.

Alternative-explanation statement: observational vitamin D associations should not be interpreted as evidence of independent host susceptibility unless studies demonstrate that the association persists after rigorous adjustment for clustered health behaviors, socioeconomic context, season/outdoor exposure, adiposity, inflammatory burden, and established oral health protection. Vitamin D may function as a risk-environment marker as well as, or instead of, a causal modifier. Table 2 summarizes the structured evidence-certainty assessment used to interpret the review findings. The certainty rating refers to confidence in the specific inference relevant to this review, not to the importance of the topic or to the existence of any biological mechanism.

**Table 2:** Structured evidence–certainty assessment by evidence domain.

Evidence domain	Main evidence base	Key appraisal concerns	Certainty for review inference	Interpretation used in this review
SSB/free-sugar exposure → caries/erosion outcomes	Systematic reviews/meta-analyses, prospective cohorts, and population studies	Mostly observational data; self-reported intake; residual confounding; heterogeneous beverage definitions	Moderate for caries; low-to-moderate for erosion	Supports SSB reduction as the clearest practice-relevant conclusion; does not establish vitamin D interaction
SSB/high-sugar exposure → oral microbiome/dysbiosis	Human saliva/plaque sequencing studies and sugar-oral microbiota reviews	Often cross-sectional; site heterogeneity; small samples; diet misclassification; batch/analysis variability	Low-to-moderate	Supports ecological plausibility and associative microbiome shifts; requires longitudinal plaque-site confirmation
Vitamin D status → caries/periodontal outcomes	Older supplementation trials, observational meta-analyses, cohorts, and causal-inference studies	Assay/cutoff heterogeneity; confounding; reverse causality; proxy-marker possibility; variable fluoride and hygiene context	Low	Associations are interpreted cautiously and are not sufficient for oral-health-specific screening or supplementation
Vitamin D → oral host-defense mechanisms	VDR/cathelicidin studies, gingival epithelial models, *in vitro* and animal evidence	Indirectness to human oral microbiome outcomes; model simplification; uncertain *in vivo* magnitude and context	Moderate for biological plausibility; very low-to-low for clinical translation	Used only as mechanistic support, not as direct evidence of human microbiome modification
Vitamin D status → human oral microbiome trajectories	Sparse human microbiome studies with vitamin D measures	Few studies; limited longitudinal data; variable sampling sites and confounder control	Very low/insufficient	Identified as a major evidence gap
SSB × vitamin D effect modification	Few or no direct human studies jointly measuring SSB, 25(OH)D, plaque microbiome, and outcomes	Absence of direct moderation models; no replicated longitudinal evidence	Very low/insufficient	Central hypothesis remains research-facing and falsifiable, not established
Vitamin D as proxy marker of broader risk environment	Cross-disciplinary evidence on determinants of 25(OH)D and oral health risk clustering	Indirect evidence; residual confounding; difficult separation from biological effects	Low-to-moderate	Maintained as an alternative explanation alongside biological susceptibility

### 6.1 SSBs and oral microbiome shifts: implications for caries and gingival outcomes

Across age groups, epidemiologic evidence supports the use of SSBs as a clinically meaningful form of caries exposure. Meta-analyses of observational studies link higher SSB intake with increased caries risk and greater odds of erosive tooth wear, and prospective cohort studies support the temporal relationship between frequent intake and greater caries increase (21–23). The consistency of these associations aligns with broader evidence that limiting free sugars reduces caries risk at the population level and informs dietary guidance and policy approaches (15, 85).

Evidence linking SSB exposure to microbiome-defined dysbiosis is newer and more heterogeneous than clinical literature. Reviews of high-sugar diets and oral microbiota generally report reduced diversity/resilience and enrichment of acidogenic or aciduric functional profiles. However, studies often rely on self-reported diet, use variable case definitions, and differ in sequencing platforms, bioinformatic pipelines, and confounder adjustment (27, 67, 68). Ecological theory suggests that site-specific supragingival plaque is the most relevant compartment for caries development, yet many studies use saliva or mouthwash samples for feasibility, which may dilute plaque-specific signals and contribute to inconsistent taxonomic findings (10, 86). Overall, the microbiome evidence base is dominated by observational (largely cross-sectional) sequencing studies and should be interpreted as associative rather than causal.

Recent research illustrates emerging patterns. Population-based analyses of the salivary microbiome have revealed lower microbial richness and distinct compositional profiles among individuals with high levels of sugar beverage consumption (87). Metagenomic studies of plaques in adolescents have indicated that microbial composition and function may modulate the extent to which sugar intake translates into caries, suggesting a mediating or modifying role for the microbiome in the sugar-caries pathway (64). Overall, the evidence for SSBs is strongest for clinical outcomes, whereas microbiome data provide supportive (but not yet definitive) evidence for a dysbiosis mechanism linking repeated sugar beverage exposure to disease. These microbiome findings are primarily derived from cross-sectional salivary profiling and smaller plaque metagenomic studies; longitudinal plaque-site studies are needed to confirm directionality and clinical relevance.

### 6.2 Vitamin D status and its association with caries and periodontal outcomes

Evidence linking vitamin D to oral disease is suggestive but generally less definitive than evidence for SSBs. In terms of caries, meta-analyses of older controlled supplementation trials suggest a protective effect of vitamin D supplementation, although many studies are older and vary in dosing, cointerventions (e.g., calcium), and diagnostic standards (88). More recent meta-analyses of observational studies, including dose–response syntheses, have reported associations between lower 25(OH)D levels and higher odds of caries, but heterogeneity and confounding remain substantial, and findings differ by age group and context (89–92).

With respect to periodontal outcomes, meta-analyses and large observational studies tend to report lower circulating 25(OH)D levels among individuals with periodontitis than among controls (93). However, Mendelian randomization analyses do not provide evidence of a substantial causal effect of genetically proxied 25(OH)D on periodontitis risk, highlighting the potential roles of confounding and reverse causality in observational associations (93, 94). Interventional studies and contemporary systematic reviews suggest that any adjunctive benefit of vitamin D supplementation during non-surgical periodontal therapy (NSPT) may be most apparent among individuals who are vitamin D deficient at baseline and achieve meaningful repletion (95). In terms of both caries and periodontal outcomes, vitamin D is most plausibly framed as a susceptibility factor that may influence risk gradients rather than as a primary causal agent. Notably, because 25(OH)D can decrease during inflammatory and acute-phase responses, the lower 25(OH)D observed in periodontitis cohorts may partially reflect the inflammatory burden rather than a causal antecedent (reverse causality). Interpretation of vitamin D “status” across studies is complicated by two related sources of heterogeneity:

1. analytical variability in 25(OH)D measurement across assays and laboratories;
2. Differences among guideline bodies regarding serum concentrations used to define deficiency or sufficiency.

For example, the Institute of Medicine concluded that serum 25(OH)D concentrations of approximately 20 ng/mL (50 nmol/L) meet the requirements of most individuals, whereas the Endocrine Society clinical practice guidelines apply higher cutoffs for defining insufficiency and sufficiency (46, 47). In parallel, documented assay variation can shift measured 25(OH)D concentrations and change how individuals are classified relative to a threshold (48–50). Accordingly, vitamin D findings are interpreted primarily as within-study contrasts (e.g., lowest vs. highest category or per-unit change) and discussed in relation to each study's assay and cutoff definitions rather than as evidence for a single universal “status” threshold.

### 6.3 Vitamin D, oral microbiome, and the host–microbe interface

Direct evidence from human studies linking vitamin D status to oral microbiome composition remains limited, and the available studies are predominantly cross-sectional, often saliva-based, and heterogeneous in terms of sampling and analytic pipelines, which constrains causal inference. Mechanistic and oral-cell studies nevertheless provide biological plausibility: vitamin D-VDR signaling can induce AMPs (including cathelicidin/LL-37) and modulate inflammatory responses and pathogen handling at epithelial barriers (76, 82). In addition, gingival tissues appear to be capable of local vitamin D metabolism, which may be particularly relevant under inflammatory conditions (70, 71). In studies that incorporate vitamin D, reports of the 25(OH)D assay method, calibration, and season of blood draw are inconsistent, which may contribute to status misclassification and attenuated associations (48, 49).

Accordingly, the evidence in this subsection is intentionally separated into human microbiome evidence (Tier 3) and mechanistic host-defense evidence (Tier 4). The VDR/antimicrobial-peptide literature should be read as a plausibility anchor for host-response biology, not as evidence that 25(OH)D status changes oral microbial community composition or function in human populations. Experimental models indicate that gingival tissues can locally activate vitamin D and that vitamin D restriction can exacerbate inflammatory and bone outcomes in periodontal challenge settings (71). These findings strengthen the biological plausibility for downstream effects on tissue response and resilience, but they do not establish the direction or magnitude of vitamin D effects on oral microbiome trajectories in humans. Accordingly, the mechanistic evidence in this section is used to frame testable pathways rather than to infer clinical benefit. This distinction is important because experimental systems often isolate single pathways, cell types, pathogens, or controlled biofilm conditions, whereas human oral microbiomes are shaped simultaneously by fluoride exposure, hygiene, saliva, diet, medications, inflammation, socioeconomic context, and site-specific ecology.

### 6.4 Interaction hypothesis: vitamin D as a modifier of sugar-driven dysbiosis

A key gap is whether vitamin D affects the pathway from SSB exposure to dysbiosis and subsequent disease in humans. Few studies have explicitly tested SSB × vitamin D interactions for microbiome endpoints or for caries/gingival outcomes using moderation models. Recent human microbiome studies suggest that the microbiome can modulate the relationship between sugar intake and caries, either by buffering or amplifying sugar-related risk (14, 64). However, the vitamin D moderation hypothesis remains largely untested in human oral microbiome research; therefore, integrating vitamin D measures into future SSB microbiome studies should be framed as a research priority to evaluate effect modification rather than as a basis for current stratified prevention. Accordingly, the proposed vitamin D interaction is presented here as a hypothesis for future testing rather than an emerging conclusion. Thus, the proposed interaction occupies the speculative-extension tier (Tier 5): it is not established by the separate Tier 1 evidence for SSB-related oral outcomes or by Tier 4 mechanistic evidence for vitamin D-regulated host defenses. Demonstration of this interaction requires studies that measure all components of the pathway simultaneously.

Future research should directly assess (1) whether low 25(OH)D levels intensify the association between SSB frequency and plaque dysbiosis (upstream moderation) and (2) whether low 25(OH)D levels enhance the association between dysbiosis and clinical outcomes (downstream moderation). Longitudinal cohort studies with repeated assessments of SSB consumption, serum 25(OH)D, plaque-site microbiome profiles, and standardized fluoride and oral hygiene covariates are essential. Such study designs would enable the distinction between mediation (microbiome as a pathway) and moderation (vitamin D altering susceptibility), thereby providing a rigorous evaluation of the ecological-host susceptibility model. A decisive test of the model would require studies that jointly measure SSB exposure patterns, serum 25(OH)D levels, site-specific plaque microbiome trajectories, and incident oral outcomes over time. Until such designs are available, the vitamin D component should remain framed as a prespecified interaction to be tested rather than a causal pathway already supported by clinical evidence.

### 6.5 Evidence-practice gap and clinical applicability

Clinical evidence strongly supports reducing free-sugar exposure, particularly frequent SSB intake, as an effective strategy to prevent caries and limit ecological pressures that promote dysbiosis. In contrast, the hypothesis that vitamin D influences the SSB–microbiome–disease axis remains speculative in humans. Direct oral microbiome studies are limited, and most mechanistic evidence is derived from *in vitro* and animal models, which do not reliably predict outcomes in diverse human populations with varying fluoride exposure, hygiene practices, and dietary patterns. Therefore, the conceptual model is intended to inform research questions and study design rather than to advocate for vitamin D supplementation as an oral microbiome intervention. In the clinic, the assessment and correction of vitamin D deficiency should adhere to established medical guidelines and patient-specific indications. Any oral health-specific screening or supplementation strategies require validation in contemporary human cohorts and clinical trials that assess plaque site microbiomes and clinically meaningful outcomes.

#### 6.5.1 Boundary of translation

To avoid overstatement, practice and policy implications are separated by evidence strength: (1) reducing frequent SSB exposure is the most defensible oral health message because it is supported by population and clinical outcome evidence; (2) encouraging water or other context-appropriate non-sugary substitutions can be framed as a sugar-exposure reduction strategy, not as a proven microbiome-restoration intervention; and (3) vitamin D testing, supplementation, or fortification should not be recommended specifically for prevention of sugar-driven oral dysbiosis unless future human studies demonstrate benefit. Adequate vitamin D remains clinically important for general health, but oral health-specific claims should be regarded as exploratory. In practical terms, mechanistic confidence should not be translated into clinical confidence. The review therefore treats experimental VDR, antimicrobial-peptide, epithelial, and animal findings as explanatory backgrounds while preserving practice-facing conclusions for human outcome evidence.

## 7 Confounding, clustering, and equity

Interpreting links among SSB exposure, oral dysbiosis, and vitamin D status requires separating potential causal effects from confounding, coexposures, and upstream social determinants. Because these factors commonly cluster (e.g., beverage environments, diet quality, preventive care, and sunlight exposure), associations, especially from cross-sectional microbiome studies, should be interpreted cautiously as potentially non-causal (1, 96).

### 7.1 Confounding in SSB–microbiome–oral outcome research

Associations between SSB intake and dental caries are supported by meta-analyses and longitudinal studies (15, 24). However, SSB consumption is correlated with total free-sugar intake, fluoride exposure, oral hygiene, and access to care, which are factors that can also shape oral microbial profiles. Accordingly, reported SSB–microbiome associations should be treated as primarily associative unless studies align plaque-site sampling with standardized preanalytic procedures and adjust for a core set of behavioral and preventive covariates (10, 12, 21, 57, 67, 68, 97).

### 7.2 Confounding in vitamin D oral outcome research

Serum 25(OH)D reflects multiple determinants (season/outdoor time, supplementation, adiposity, and lifestyle factors) that can also be related to oral health and SSB intake through shared social contexts (46). Therefore, observational vitamin D–oral outcome associations require careful adjustment and, where possible, stratified analyses to evaluate whether relationships differ by baseline risk or context (5, 6). Reverse causality and inflammatory feedback may bias observed associations between 25(OH)D and periodontal outcomes. Circulating 25(OH)D can decrease during systemic inflammatory responses, potentially acting as a negative acute-phase reactant through mechanisms such as altered vitamin D–binding protein and redistribution. Because periodontitis is a chronic inflammatory condition, active disease may contribute to lower measured 25(OH)D levels, meaning that disease status could influence exposure classification. Accordingly, cross-sectional findings should be interpreted with caution. Longitudinal studies incorporating repeated 25(OH)D measurements alongside inflammatory markers (e.g., C-reactive protein: CRP) are needed to better distinguish causal effects from consequences of inflammation (30, 98).

Low 25(OH)D may represent as a proxy marker of broader risk environments rather than an independent determinant of oral susceptibility. Reduced vitamin D status often cooccurs with poorer diet quality, higher adiposity, limited supplement or fortified food use, reduced outdoor activity, socioeconomic disadvantage, and chronic systemic inflammation. These correlated factors can confound observed associations because they independently influence oral health through differences in SSB intake, oral hygiene, fluoride exposure, access to dental care, salivary function, and periodontal inflammation. Consequently, associations between 25(OH)D and the oral microbiome should be interpreted cautiously, as they may reflect both biological effects and residual confounding from clustered risk exposures.

### 7.3 Exposure clustering: SSBs rarely act alone

SSB-intake frequently co-occurs with broader dietary patterns (e.g., ultra-processed foods and snacking) and with host factors relevant to vitamin D status and periodontal risk (e.g., smoking in adults, xerostomia-inducing medications, and metabolic health) (15, 30, 67). This clustering can make single-exposure interpretations misleading; Table 3 highlights a pragmatic set of covariates and design features that can improve interpretability.

**Table 3:** Key confounders and how future studies should control them.

Confounder/cluster factor	Why it matters (bias pathway)	What to measure (minimum)	How to control (design + analysis)	Key refs
Fluoride exposure	Strong protection against caries; socially patterned; correlated with dental care access and hygiene	Water fluoridation status; fluoride toothpaste use (frequency); professional fluoride/sealants	Restrict or stratify by fluoride access; adjust in models; report effect modification	(1, 15, 21)
Oral hygiene behaviors	Alters plaque mass, pH dynamics, and microbiome; correlated with SES and diet	Brushing frequency; interdental cleaning; mouthwash use; last brushing time	Standardize presampling instructions; adjust/stratify; sensitivity analyses	(5)
Baseline oral health status	Disease state powerfully shapes microbiome and outcomes; can reverse-cause diet change	Caries indices (DMFT/ICDAS); gingival index/BOP; periodontal screening where age-appropriate	Match cases/controls on oral status; include as covariate; analyze incident outcomes longitudinally	(10)
Total free sugars (foods + drinks)	SSBs may proxy overall sugar exposure	24 h recalls/FFQ; total free sugar; snack frequency	Model SSB independently of total sugar; dietary pattern methods	(15)
Overall diet quality/ultra-processed diet pattern	Coexposures cluster with SSBs and affect micronutrient adequacy	Diet quality index; UPF proportion; fruit/veg intake	Use pattern-based covariates; propensity scores; DAG-informed adjustment	(6)
Socioeconomic position (SEP)	Drives both exposure and protection (marketing, access to care/fluoride, food choices)	Education, income, neighborhood deprivation	Multilevel models; stratified analyses; test SEP as effect modifier	(57)
Dental care access & preventive services	Preventive care reduces outcomes; access correlates with SEP	Dental visits; sealants; preventive counseling	Adjust for access; stratify; incorporate health-system variables	(5)
Antibiotic exposure & medications	Alters microbiome composition; medications can change saliva flow and pH	Antibiotics in past 2–3 months; chronic meds; xerostomia symptoms	Exclude recent antibiotics; include time-since-antibiotics; adjust for xerostomic meds	(67)
Salivary flow/xerostomia	Low saliva increases acid persistence; influences microbiome and caries risk	Flow rate (if possible); xerostomia questionnaire; hydration status	Measure and adjust; stratify by xerostomia; record time of day	(67)
Smoking/alcohol (adults)	Strongly affects periodontal inflammation and oral microbiome; correlated with SEP	Smoking status; alcohol frequency	Restrict to non-smokers or adjust; stratify; sensitivity analyses	(98)
BMI/metabolic health	Correlates with SSB intake and vitamin D status; affects inflammation	BMI; diabetes status; CRP (optional)	Adjust; stratify; mediation vs. confounding discussion	(69)
Metabolic/genetic background	May correlate with subgingival microbiota and inflammatory susceptibility beyond SSB or vitamin D exposure	Metabolic phenotype; ancestry; host genotyping if available	Adjust or stratify as exploratory modifiers; avoid causal overinterpretation	(37)
Season, sun exposure, supplementation (vitamin D determinants)	Confounds vitamin D associations; linked to behaviors and SEP	Season of blood draw; outdoor time proxy; supplement use; 25(OH)D	Adjust; include interaction terms; repeated measures across seasons	(30)
Microbiome technical factors (batch effects)	Primer choice, platform, storage, extraction introduce systematic bias	Sample site; storage time; extraction kit; 16S region; sequencing run	Standardize pipeline; randomize samples across batches; include batch covariates	(67)
Vitamin D as proxy indicator of risk environment	Low 25(OH)D may reflect clustered diet, outdoor time, adiposity, inflammation, supplement access, SEP, and preventive-care conditions rather than independent susceptibility	Season/outdoor time; diet quality; supplement/fortified-food use; BMI/adiposity; CRP or inflammatory markers; SEP/neighborhood context; dental-care access	Use DAGs; compare minimally vs. fully adjusted models; report attenuation; conduct stratified/sensitivity analyses; avoid causal language when residual clustering remains	(4, 30, 99, 100)

SEP, socioeconomic position; BOP, bleeding on probing; UPF, ultra-processed foods; DAG, directed acyclic graph.

This clustering has direct implications for interpretation; attenuation or disappearance of vitamin D associations after adjustment for socioeconomic position, diet quality, adiposity, inflammation, fluoride/hygiene, and dental-care access would support a proxy-marker explanation, whereas robust associations across well-characterized contexts would strengthen the case for independent susceptibility. Both outcomes are informative and should be reported transparently. Metabolic dysfunction and host genetic background should therefore be considered not only as distal risk markers but also as possible correlates of microbial community structure. Studies by Nibali et al. highlight that the subgingival microbiota may covary with metabolic phenotypes and metabolic-associated host genetic variants, supporting careful measurement of metabolic status and, where feasible, the genomic context in future oral microbiome studies (37, 55).

### 7.4 Equity: shared structural determinants drive unequal risk

Oral diseases are socially patterned: disadvantage often concentrates harmful beverage exposures and reduced access to protective resources such as fluoride, preventive care, and healthier food environments (4). Frameworks such as the common risk factor approach and the London Charter support upstream prevention, but within the present evidence base, those implications should focus on reducing sugar exposure and improving established protections rather than proposing vitamin D screening as an oral health policy. The observation that low vitamin D status and high SSB exposure may cooccur in some populations should be used mainly to improve confounding control and equity-sensitive study design (4, 6, 57, 69).

### 7.5 Implications for future studies

Future studies should prioritize a small set of high-leverage design features: detailed SSB pattern measurement, plaque-site sampling when caries mechanisms are targeted, standardized reporting of vitamin D assays/cutoffs and key determinants (season, adiposity, supplements), and adjustment for fluoride exposure, hygiene, baseline disease, antibiotics, and socioeconomic context. Where feasible, analyses should test prespecified interaction models [SSB × 25(OH)D] and report results stratified by baseline risk and context.

## 8 Research gaps and future directions

Key gaps are related to aligning exposure and sampling measures with oral ecology, improving the comparability of vitamin D assessment, and generating human evidence that explicitly tests whether vitamin D affects SSB–microbiome–disease relationships (20, 21).

### 8.1 Measurement and sampling gaps

*“*Sugar dose” is not the same as “ecological pressure”; SSB exposure is often captured with broad self-report measures and rarely resolves frequency, timing, and beverage subtype, features that may be more informative than total volume alone. Future studies should combine quantitative intake with pattern measures (e.g., between-meal/bedtime intake and sipping) and, where feasible, use repeated or technology-assisted assessment to reduce misclassification (15). Many studies profile saliva for feasibility, but plaque sampled from lesion-prone sites is more closely aligned with caries mechanisms; site heterogeneity can therefore dilute associations (10). A practical next step is to prioritize plaque-site sampling in hypothesis-testing studies, standardize key preanalytic metadata, and complement taxonomic profiles with functional measures when available (64, 67).

Across studies, “vitamin D status” is not standardized: 25(OH)D cutoffs, assays, calibration, and sampling timing vary, and intraassay variability can change classification around commonly used thresholds (46). Synthesis should therefore emphasize within-study contrasts and transparent reporting of the assay platform, units, season, and both continuous and categorical 25(OH)D analyses (49, 70, 78). Direct human evidence linking 25(OH)D to oral microbiome profiles remains limited; future cohorts and trials should measure 25(OH)D repeatedly (where feasible), collect plaque-site microbiomes, and evaluate interaction models while accounting for inflammatory status to reduce reverse causality bias (48, 50). Given the possibility that inflammation-related processes can lower 25(OH)D, future cohort and intervention studies should also report inflammatory status (e.g., CRP) and consider the timing of vitamin D sampling relative to active periodontal inflammation to mitigate reverse causality bias (100, 101).

### 8.2 Causal designs and analytic priorities: testing moderation and mediation

Few studies formally test moderation or mediation in this domain. Longitudinal designs with repeated SSB exposure, 25(OH)D, and microbiome measures are needed to evaluate whether vitamin D changes the strength of the SSB → dysbiosis association or the dysbiosis → outcome association; transparent reporting of microbiome processing and analysis improves comparability across studies (68, 97). To distinguish an independent vitamin D modifier from a proxy-marker explanation, future analyses should prespecify directed acyclic graphs, report sequential adjustment models, and examine whether 25(OH)D associations persist after accounting for social context, diet quality, adiposity/metabolic status, inflammatory markers, fluoride/hygiene, and access to dental care. Negative-control or triangulation approaches may also help identify residual confounding.

### 8.3 Intervention and equity priorities

Intervention evidence linking SSB reduction to oral microbiome change is limited. Pragmatic beverage substitution trials and natural policy experiments should therefore evaluate, rather than assume, microbiome and clinical effects; vitamin D supplementation studies should be limited to deficiency correction or established medical indications, with oral outcomes and plaque microbiomes treated as exploratory secondary endpoints (58–60). Equity-focused designs are essential because exposure and protection clusters: studies should measure socioeconomic context, fluoride access, and preventive care access and assess whether interventions reduce or widen inequalities (4–6).

### 8.4 Strengths and limitations

Strengths: (1) explicit reframing as a narrative-conceptual review with structured evidence mapping rather than an effect-estimating systematic review; (2) transparent documentation of literature identification and selection using a PRISMA-ScR-style flow diagram; (3) integration of evidence across diet, oral microbiome ecology, vitamin D biology, metabolic context, and oral health outcomes; and (4) explicit treatment of interpretive challenges, including limited direct human microbiome evidence, heterogeneity in vitamin D assessment, reverse causality, confounding, and equity-related exposure clustering.

Limitations: (1) This is a narrative, not a formal systematic or registered scoping review, and does not provide pooled estimates; (2) the SSB × vitamin D moderation hypothesis remains sparsely tested in humans, especially with plaque-site microbiome outcomes; (3) diet–microbiome studies are heterogeneous in exposures, sampling, and analytic methods; and (4) although domain-level evidence certainty is assessed, a full study-level risk-of-bias evaluation was not feasible. Accordingly, conclusions are graded by evidence stream and certainty rather than framed as definitive. The conceptual model also relies on triangulation across studies, and the proposed vitamin D moderation pathway has not been validated as a unified human mechanism.

## 9 Conclusion

This narrative synthesis offers an integrative framework rather than pooled estimates. SSB intake is consistently linked to caries (less so to erosion), and microbiome evidence suggests less resilient communities with higher exposure, although heterogeneity across studies warrants cautious interpretation. Vitamin D is a plausible susceptibility factor; however, current evidence that circulating 25(OH)D levels shape oral or gut microbiome trajectories is limited and warrants validation in longitudinal studies (102). Priorities remain in terms of reducing SSB exposure and reinforcing established protection (103); vitamin D care should follow guidelines, with oral benefits considered exploratory. Low 25(OH)D may reflect broader risk states rather than causality. This review does not establish vitamin D as a modifier of sugar-driven oral dysbiosis; rather, it presents a transparent, falsifiable model to guide future human studies and limit overinterpretation of indirect evidence. It also separates evidence-based prevention (SSB reduction and standard oral care) from hypotheses requiring prospective validation. Accordingly, evidence from VDR signaling, antimicrobial peptides, epithelial responses, and animal periodontal models should be viewed as mechanistic support rather than as evidence that vitamin D affects oral microbial ecology or SSB-related disease risk in humans. Moreover, evidence linking metabolic dysfunction and host genetics to the oral microbiome suggests that vitamin D is one of several host susceptibility factors that influence oral microbial ecology rather than the sole systemic determinant.

## Data Availability

The original contributions presented in the study are included in the article/Supplementary Material, further inquiries can be directed to the corresponding author/s.
